# Combating head and neck cancer metastases by targeting Src using multifunctional nanoparticle-based saracatinib

**DOI:** 10.1186/s13045-018-0623-3

**Published:** 2018-06-20

**Authors:** Liwei Lang, Chloe Shay, Yuanping Xiong, Parth Thakkar, Ron Chemmalakuzhy, Xuli Wang, Yong Teng

**Affiliations:** 1 Department of Oral Biology, Dental College of Georgia, Augusta University, Augusta, GA USA; 2 Department of Pediatrics, Emory Children’s Center, Emory University, Atlanta, GA USA; 3 Department of Biology, College of Science and Mathematics, Augusta University, Augusta, GA USA; 4 Department of Radiology and Imaging Sciences, School of Medicine, University of Utah, 201 Presidents Cir, Salt Lake City, UT 84112 USA; 5 Georgia Cancer Center, Department of Biochemistry and Molecular Biology, Medical College of Georgia, Augusta University, Augusta, GA USA; 6 Department of Medical Laboratory, Imaging and Radiologic Sciences, College of Allied Health, Augusta University, 1120 15th Street, Augusta, GA 30912 USA

**Keywords:** Src, Saracatinib, Nanoparticles, HNSCC, Metastasis

## Abstract

**Background:**

Inhibition of metastasis of head and neck squamous cell carcinoma (HNSCC) is one of the most important challenges in cancer treatment. Src, a non-receptor tyrosine kinase, has been implicated as a key promoter in tumor progression and metastasis of HNSCC. However, Src therapy for HNSCC is limited by lack of efficient in vivo delivery and underlying mechanisms remain elusive.

**Methods:**

Src knockdown cells were achieved by lentiviral-mediated interference. Cell migration and invasion were examined by wound healing and Transwell assays. Protein levels were determined by Western blot and/or immunohistochemistry. The Src inhibitor saracatinib was loaded into self-assembling nanoparticles by the solvent evaporation method. An experimental metastasis mouse model was generated to investigate the drug efficacy in metastasis.

**Results:**

Blockade of Src kinase activity by saracatinib effectively suppressed invasion and metastasis of HNSCC. Mechanistic assessment of the drug effects in HNSCC cells showed that saracatinib induced suppression of Src-dependent invasion/metastasis through downregulating the expression levels of Vimentin and Snail proteins. In tests in mice, saracatinib loaded into the novel multifunctional nanoparticles exhibited superior effects on suppression of HNSCC metastasis compared with the free drug, which is mainly attributed to highly specific and efficient tumor-targeted drug delivery system.

**Conclusions:**

These findings and advances are of great importance to the development of Src-targeted nanomedicine as a more effective therapy for metastatic HNSCC.

**Electronic supplementary material:**

The online version of this article (10.1186/s13045-018-0623-3) contains supplementary material, which is available to authorized users.

## Background

More than 90% of tumors in the head and neck are squamous carcinomas (HNSCC) that arise in the paranasal sinuses, nasal cavity, oral cavity, pharynx, and larynx [1]. About two-thirds of patients with HNSCC present with advanced-stage disease (stages III and IV), and the high rate of metastasis is highly associated with a poor 5-year survival rate [2, 3]. Despite significant improvements in multiple-modality therapy with surgery, chemotherapy, and radiation, long-term survival rates in patients with advanced-stage HNSCC have not increased significantly in the past few decades [4]. The poor clinical outcomes reveal an obvious and urgent need to develop more effective and tolerated treatments against HNSCC, especially for aggressive tumors.

Modern research is now focusing on seeking specific molecular targets involved in the development and procession of cancer in an attempt to develop more officious and selective treatments. Src, a member of Src family of non-receptor tyrosine kinases (SFKs), is often activated by direct or indirect interaction with receptor tyrosine kinases (RTK), such as epidermal growth factor receptor (EGFR), platelet-derived growth factor receptor (PDGFR), fibroblast growth factor receptor (FGFR), and insulin-like growth factor 1 receptor (IGF-1R), as well as G-protein-coupled receptors (GPCRs), cytokines, integrins, and others [5]. It appears that Src acts as a critical molecular switch in regulating signal transduction for many fundamental cellular processes by a diverse set of cell surface receptors in the context of a variety of cellular environments. Overexpression and hyperactivation of Src have been found in a wide variety of human cancers, including HNSCC [5, 6]. Additionally, the extent of increased Src activity often correlates with malignant potential and patient survival [7]. Multiple signaling pathways converge on Src activation to epithelial-mesenchymal transition (EMT) phenotypic features to promote tumor cell metastasis [8]. In mice models of breast cancer, inhibition of Src kinase activity can improve survival through suppressing metastasis [9]. In HNSCC, Src is activated following EGF stimulation and decreases cell migration and invasion in treatment with Src inhibitors [10, 11]. The involvement of Src in tumor progression and metastasis has generated considerable interest in Src as a therapeutic strategy to treat metastatic disease.

Src-targeting agents, including dasatinib and saracatinib (AZD0530), are currently in clinical development for patients with solid tumors. Dasatinib, a potent oral tyrosine kinase inhibitor which targets Src and other several kinases [7], has shown a marked efficacy in patients with chronic myeloid leukemia (CML) as first-line treatment [12]. The capacity of dasatinib to block migration and invasion without affecting proliferation and survival is demonstrated in human melanoma cells [13]. Dasatinib is also reported to suppress migration and invasion of HNSCC cells, coupled with the inhibition of Src and downstream mediators of cell adhesion, such as focal adhesion kinase (FAK) [11]. Dasatinib as a single agent has modest clinical activity with liver failure on many types of solid tumors, including non-small cell lung cancer, prostate cancer, and breast cancer [14]. Saracatinib, originally developed by AstraZeneca, is a novel anilinoquinazoline inhibiting deregulated elevated Src kinase activity in a wide range of cancer cells, such as colorectal, ovary, prostate, and breast cancer [7, 15–17]. Several preclinical reports suggest that saracatinib has potent anti-migratory and anti-invasive effects in endocrine-resistant breast cancer cells [18] and significantly suppressed the metastatic nature of bladder cancer in a murine model [19]. Although saracatinib was evaluated in phase I/II clinical trials for advanced stage HNSCC and other various types of cancer [20], the anticancer efficacy was not sufficiently promising to justify continued accrual to active trials. Therefore, developing a novel saracatinib-based strategy would open a new avenue for Src-targeted therapy.

Physicochemical and pharmacokinetic profiles of anticancer drugs render optimal delivery challenging. Moreover, distribution, biotransformation, and clearance of anticancer drugs in the body must be overcome to deliver therapeutic agents to tumor cells in vivo [21]. Nanoparticles (NPs) have shown promise as both drug delivery vehicles and direct anticancer systems, based on the quantum properties and the ability to carry and absorption [22]. Most solid tumors possess unique pathophysiological characteristics that are not observed in normal tissues or organs (e.g., extensive angiogenesis, low pH and hypoxia), which greatly increase production of a number of the tumor site-specific delivery of NPs. Numerous studies have shown that both tissue and cell distribution profiles of anticancer drugs can be controlled by their entrapment in NPs [23, 24].

In the present study, we show that Src is one of the most targetable molecules involved in invasion and metastasis of HNSCC, and saracatinib can significantly suppress the invasive and metastatic phenotype through inhibiting Src kinase activity and its mediated metastatic signaling in HNSCC cells. We also designed and synthesized novel multifunctional NPs for selective release of saracatinib into head and neck tumor cells and evaluated the anti-tumor efficacy and efficiency of saracatinib-loaded NPs (Nano-sar) in mice. Our studies reveal that Nano-sar has superior anticancer effects than the free drug through suppressing head and neck tumor metastasis more efficiently. The tumor site-specific delivery of NPs, especially with the use of saracatinib, would be straightforwardly extended from HNSCC to other types of solid tumors.

## Methods

### Cell lines and standard assays

HNSCC cell lines HN6, HN8, and HN12 were maintained in Dulbecco’s Modified Eagle’s Medium (DMEM) containing 10% fetal bovine serum as previously described [25, 26]. HN6 was derived from tongue squamous cell carcinoma. HN8 and HN12 were derived from the metastatic lymph node site from oral cavity and tongue squamous cell carcinoma, respectively. The cell passage number less than 10 was used for experiments. Cell proliferation was determined by CellTiter 96® AQueous One Solution Cell Proliferation Assay (MTS) (Promega, Madison, WI), and invasion was determined by Transwells (BD biosciences, San Jose, CA) with 8-μm pore size filters covered with Matrigel. Transfection and infection, colony formation, and scratch-wound healing were carried out as previously described [7, 27–29].

### Constructs, reagents, and antibodies

pLKO.1 lentiviral vectors harboring short hairpin RNAs (shRNAs) targeting Src or green fluorescent protein (GFP) were obtained from Open Biosystems (Huntsville, AL). Saracatinib and dasatinib were purchased from Selleckchem (Houston, TX). For Western blot, antibodies that recognize p-Src (Tyr416) and Src were purchased from Cell Signaling Technology (Beverly, MA). β-Actin antibody was purchased from Sigma-Aldrich (St Louis, MO). Epithelial-Mesenchymal Transition (EMT) Antibody Sampler Kit (#9782) and Tight Junction Antibody Sampler Kit (#8683) were purchased from Cell Signaling Technology (Beverly, MA).

### Western blot assay

The protein levels for the biomarkers were semi-quantified by Western blot analysis as previously described [29, 30]. Electrophoresis was performed on 10% SDS-PAGE gel, and the proteins were transferred to nitrocellulose membrane. The membranes were incubated with the primary antibodies overnight at 4 °C and with secondary antibody for 1 h at room temperature. The antigen-antibody complexes were then visualized using Clarity™ Western ECL Substrate (Bio-Rad, Hercules, CA). The protein bands were quantified by densitometry analysis.

### Solid-phase peptide synthesis

Synthesis of the peptide was carried out using the Fmoc strategy manually in a glass reaction vessel fitted with a sintered glass frit using 2-chlorotritylchloride. Coupling reactions were performed manually by using 2 equiv. of N-Fmoc-protected amino acid (relative to the resin loading) activated in situ with 2 equiv. of PyBOP and 4 equiv. of diisopropylethylamine (DIPEA) in DMF (10 mL/g resin). The coupling efficiency was assessed by Kaiser test. N-Fmoc protecting groups were removed by treatment with a piperidine/DMF solution (1:4) for 10 min (10 mL/g resin). The process was repeated three times and the completeness of deprotection verified by UV absorption of the piperidine washings at 301 nm. Synthetic linear peptides were recovered directly upon acid cleavage. Before cleavage, the resin was washed thoroughly with methylene chloride. The linear peptides were then released from the resin by treatments with a solution of acetic acid/trifluoroethanol/methylene chloride (1:1:8, 10 mL/mg resin, 2 × 30 min). Hexane (5–10 volumes) was added to the collected filtrates, and the crude peptides were isolated after concentration as white solids. The residue was dissolved in the minimum of methylene chloride, and diethyl ether was added to precipitate peptides. Then, they were triturated and washed three times with diethyl ether to obtain crude materials. Peptide was further purified by preparative high-performance liquid chromatography (HPLC) prior to conjugation.

### Synthesis of the polymeric drug carrier

Linear-dendritic mPEG-BMA4 was synthesized according to a method in literature [31, 32]. Under a nitrogen atmosphere, branched mPEG-BMA4 (1 equv. based on amino group), peptide (Ac-K(Boc) GFLG-OH, 1 equv.), HBTU (1 equv.), and HOBT (1 equv.) were added into a round flask and dissolved in anhydrous DMF. Then, DIPEA (2 equv.) was added dropwise under ice bath. The solution was stirred in ice bath for 30 min and at room temperature for 48 h. The solution was dialyzed against deionized water using dialysis membrane (MWCO = 2000). The final product was obtained via lyophilization.

### Saracatinib-loading into NPs

Hydrophobic saracatinib was loaded into the NPs by the solvent evaporation method as described in literature [33, 34]. Briefly, drug (1.0 mg) and amphiphilic polymer (10 mg) were first dissolved in anhydrous chloroform/methanol (1/1) in a 10 mL round bottom flask. The solvent mixture was evaporated under vacuum to form a thin film. PBS buffer (1 mL) was added to re-hydrate the thin film, followed by 30 min of sonication. The unloaded drug was removed by running the NP solutions through centrifugal filter devices (MWCO: 3.5 kDa, Microcon®). The saracatinib-loaded formulation on the filters were recovered with PBS.

### Characterization of NPs

The amount of drug loaded in the NPs was analyzed on a HPLC system (Agilent 1200 LC, Santa Clara, CA). The drug loading was calculated according to the calibration curve between the HPLC area values and concentrations of drug standard. The loading efficiency was defined as the ratio of drug loaded into NPs to the initial drug content. The size and size distribution of Nano-sar were measured by dynamic light scattering (DLS) instrument for three times with an acquisition time of 30 s at room temperature.

### Drug release study

The drug release from Nano-sar was carried out in the solution with or without cathepsin B (CTSB). Cysteine solution in McIlvaine’s buffer (10 mm) was added in equal volume of enzyme stock solution and pre-incubated at 37 °C for 5 min. NPs were incubated in the buffer at 37 °C for 48 h in the presence or absence of CTSB (100 nM, pH = 5.4). A drug release control study at physiological condition (without enzyme, pH 7.4) was also performed. At pre-determined time points, the samples were withdrawn and analyzed by reversed-phase HPLC (RP HPLC) with gradient elution.

### Three-dimensional (3D) tumor spheroid invasion assay

The experiment was modified and carried out as previously described [35, 36]. Briefly, 2 × 10^4^ HN12 cells were incubated overnight to form 3D spheroid in hanging droplet in a well of an inverted round bottom 96-well plate. Then, 150 μl mixture of Matrigel: DMEM without serum (1:1 ratio) was added in the well and solidified at 37 °C, followed by adding 150 μl complete culture medium containing double doses of drugs. After 3 days, spheroids from different treatments were imaged under a microscope.

### Animal study

Six-week-old NOD.Cg*-Prkdcscid Il2rgtm1Wjl/SzJ* (NSG) mice were purchased from the Jackson Laboratory (Bar Harbor, ME, USA), and all animal experiments were approved by the Institutional Animal Care and Use Committee (IACUC) of Augusta University. To generate a metastasis model in NSG mice, 5 × 10^5^ HN12 with a luciferase reporter gene were suspended in 100 μl of PBS/Matrigel (3:1) and injected into the right flank. When mean tumor volumes reached approximately 100 mm^3^, mice were randomized to receive equal volume treatment of vehicle (sterile saline), the free drug saracatinib (20 mg/kg), or Nano-sar (at dose 10 mg/kg) by tail vein administration every other day for a total of 12 days. Tumor growth was measured externally every 4 days using vernier calipers as length × width^2^ × 0.52. Mice were imaged for bioluminescent luciferase signal by an intraperitoneal injection of D-luciferin bioluminescent substrate (Sigma-Aldrich, St Louis, MO) using a Xenogen IVIS-200 In Vivo Imaging System (PerkinElmer, Waltham, MA). When the experiment was terminated, blood was collected via ocular vein for determination of serum Alanine Transaminase (ALT/GPT), Aspartate Transaminase (AST/GOP), and creatinine. ALT and AST were measured by EnzyChrom™ Alanine Transaminase Assay Kit and Aspartate Transaminase Assay kits (BioAssay System, Hayward, CA), respectively. Serum creatinine was measured by Creatinine Assay Kit (Cayman chemical, Ann Arbor, MI). The mice were then sacrificed, and the xenografts and the major organs (heart, intestine, liver, spleen, lung, and kidney) were removed for histopathological analysis with hematoxylin-and-eosin (H&E) staining.

### Immunohistochemistry (IHC)

Paraffin-embedded xenografts were cut into 3 μm sections and mounted on slides, and IHC was performed as described previously [7, 37]. Briefly, tissue sections were blocked in 10% of normal goat serum after antigen retrieval in hot citrate buffer and were incubated with the primary antibodies against p-Src, Vimentin, and Snail, respectively. Immuno-reactivity was visualized by using the DAB Kit (Vector Laboratories, Burlingame, CA, USA) according to the manufacturers’ procedure, and images were reviewed and analyzed by a CCD camera (Olympus, Center Valley, PA). At least nine random microscopic fields were captured, and signal intensity was quantified using the Image pro-Plus6.0 software.

### Statistical analysis

Treatment effects were evaluated using one-way ANOVA at each measurement time-point. To assess the longitudinal effect of treatment, a mixed model was employed to test the overall difference across all groups as well as between each pair of groups during the whole study period. Experiments shown are the means of multiple individual points from multiple experiments (± S.D.), and *p* < 0.05 was considered as statistically significant.

## Results

### Saracatinib strongly inhibits Src kinase activity and migration in HNSCC cells

**Fig. 1:**
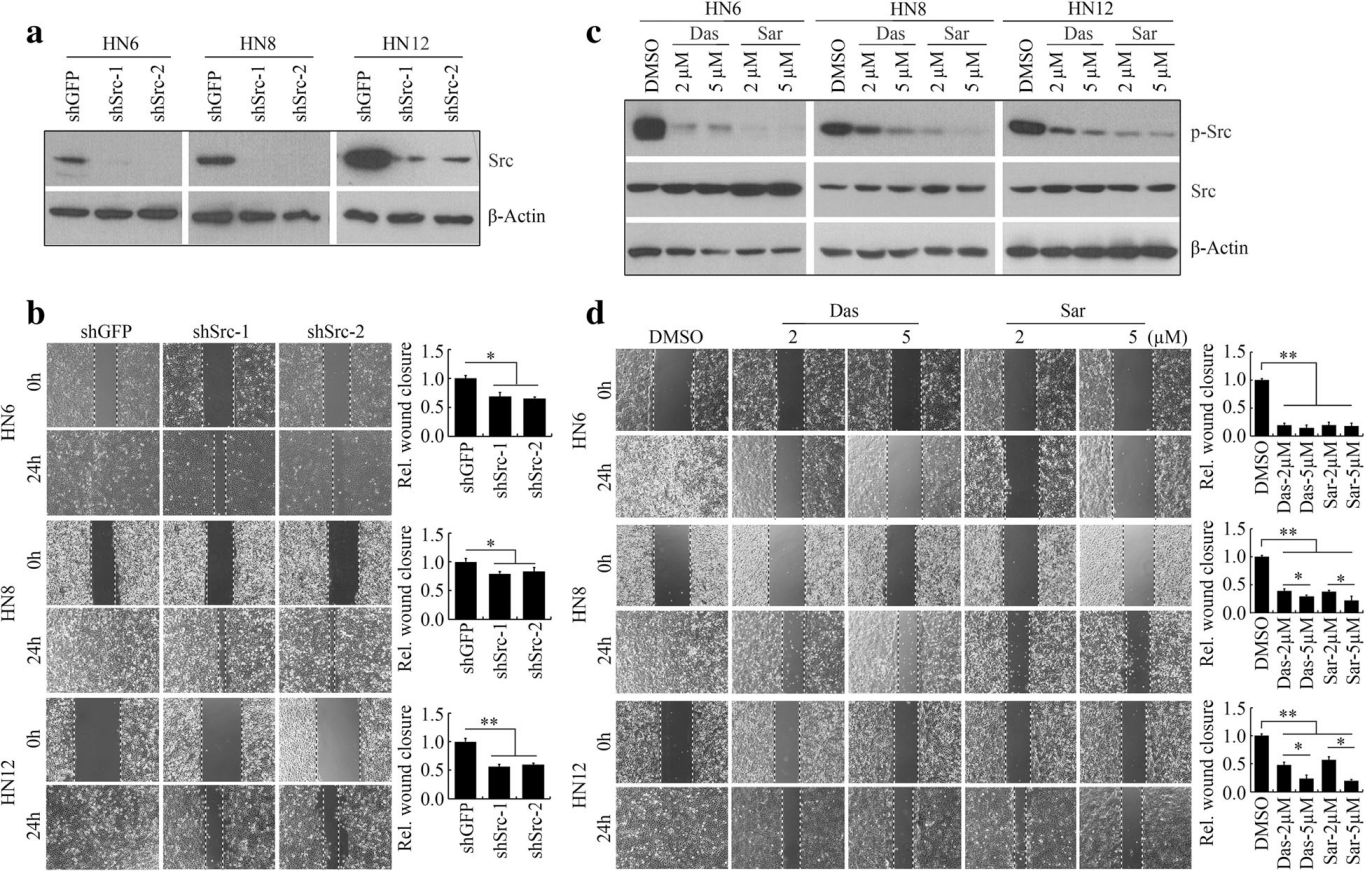
Saracatinib effectively inhibits Src phospho-activation and migration in HNSCC cells. **a** The effects of shRNAs against Src on the expression of Src protein. **b** The effects of Src knockdown on cell migration within 24 h. **c** The effects of saracatinib and dasatinib on the phosphorylation levels of Src. **d** The effects of saracatinib and dasatinib on cell migration within 24 h. **b**, **d** The representative images and quantitative data were shown in the left and right panels, respectively. *p < 0.05; **p < 0.01

To study the role of Src in cell movement, we depleted Src in high-invasive HNSCC cells (HN6, HN8, and HN12) by shRNAs. Lentivirus-mediated knockdown of Src remarkably reduced Src expression levels, leading to decreased migration compared with the control cells transfected with a shRNA against GFP (Fig. 1a, b). These results showed that loss of Src reduced migratory potential in HNSCC cells. We then treated HN6, HN8, and HN12 cells with dasatinib and saracatinib, which showed increased protein levels of Src upon drug treatment (Fig. 1c). However, dramatically decreased Src phosphorylation was observed in cells either treated with dasatinib or saracatinib (Fig. 1c). Saracatinib-induced Src inactivation was in a dose-dependent manner, and this inhibitory effect was more efficient than dasatinib at the same dosage (Fig. 1c). Moreover, a clear reduction in scratch-wound healing capability was noted in cells exposed to dasatinib or saracatinib (Fig. 1d), which was consistent with the observations in Src knockdown cells (Fig. 1b).

### Saracatinib suppresses migration of mesenchymal-like HNSCC cells by inactivating Src-dependent Vimentin/Snail signaling

**Fig. 2:**
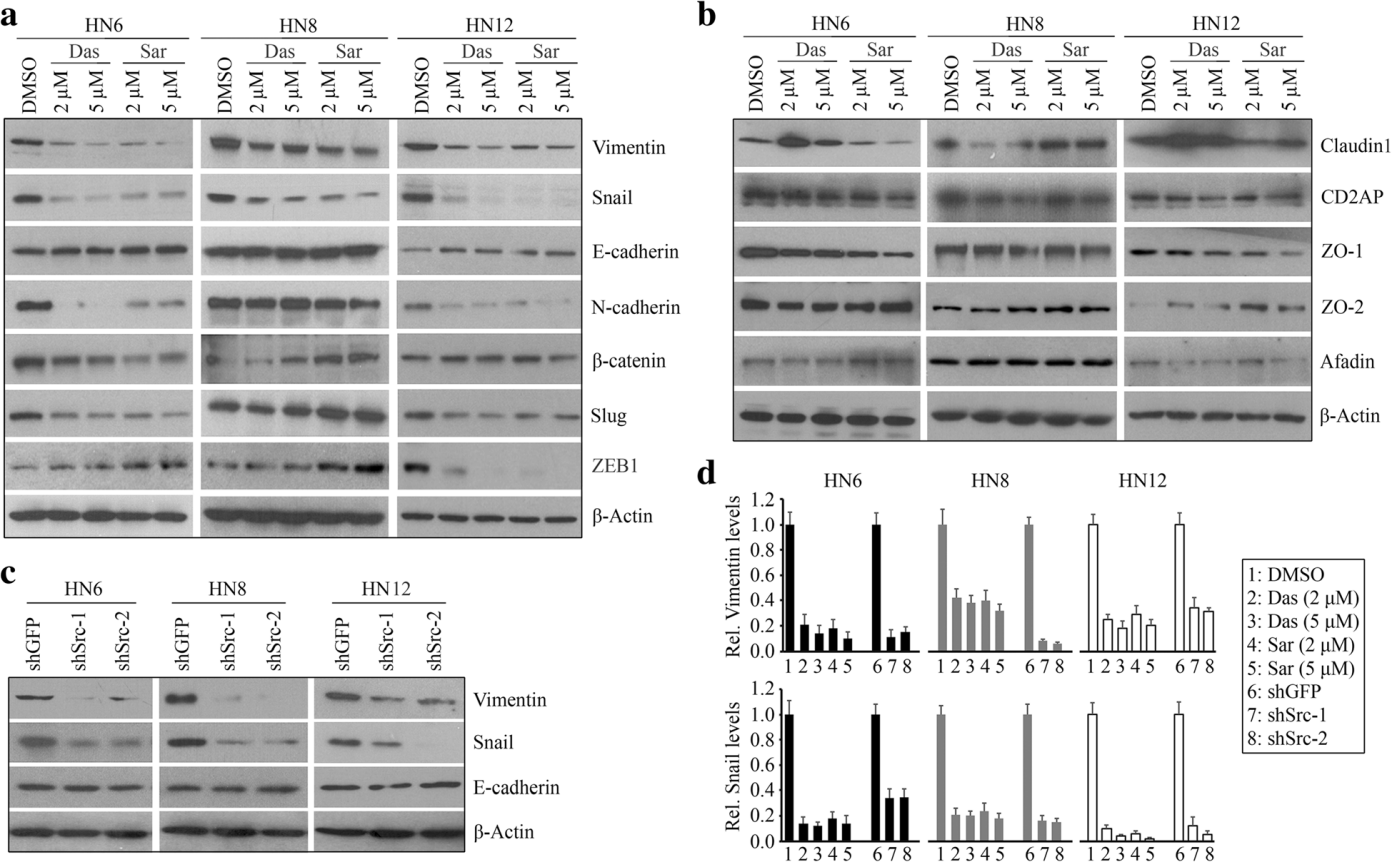
Saracatinib suppresses Src-dependent Vimentin/Snail signaling in HNSCC cells. **a** The effects of saracatinib and dasatinib on the expression of EMT-related proteins. **b** The effects of saracatinib and dasatinib on the expression of cell tight junction-related proteins. **c** The effects of Src knockdown on the expression of Vimentin, E-cadherin, and Snail proteins. **d** Quantification of relative protein levels of Vimentin and Snail among the different treatments from three independent experiments

Src has been shown to play an important role in promoting EMT [8], which often contributes to cancer cell migration and invasion. We thus assessed whether loss of Src led to a reversal of EMT in HNSCC cells. HN6, HN8, and HN12 cell lines examined in this study were mesenchymal-like cells, and they switched to epithelial-like shape when loss of Src expression (Additional file 1: Figure S1A). Similar to the observations from Src knockdown cells, cells appeared to clump together with the Src inhibitor treatment (Additional file 1: Figure S1B). To understand the mechanism involved, we determined the molecules that were mostly involved in EMT process. This analysis showed a sharp decrease in protein levels of mesenchymal marker Vimentin in the presence of Src inhibitors, which was accompanied by downregulation of Snail (Fig. 2a, d). There were no significant changes in the protein levels of epithelial marker E-cadherin protein, indicating that E-cadherin does not contribute to HNSCC cell MET induced by Src inactivation (Fig. 2a). No consistent tendency of other EMT-related proteins (N-cadherin, β-catenin, Slug, and ZEB1) was observed in three cell lines in the presence and absence of Src inhibitors, excluding their common functions in drug-induced diminishment of EMT traits in mesenchymal-like HNSCC cells (Fig. 2a). We also examined tight junction-related proteins (Caludin1, CD2AP, ZO-1, ZO-2, and Afadin), which showed cell content-dependent changes in the treatment with Src inhibitors (Fig. 2b). To study whether saracatinib and dasatinib share a common mechanism to regulate cell motility through downregulation of Src-dependent Vimentin and Snail expression, we determined these molecules in Src knockdown cells. Consistently, knockdown of Src was associated with decreased Vimentin and Snail levels (Fig. 2c, d).

### Synthesis and characterization of Nano-sar

**Fig. 3:**
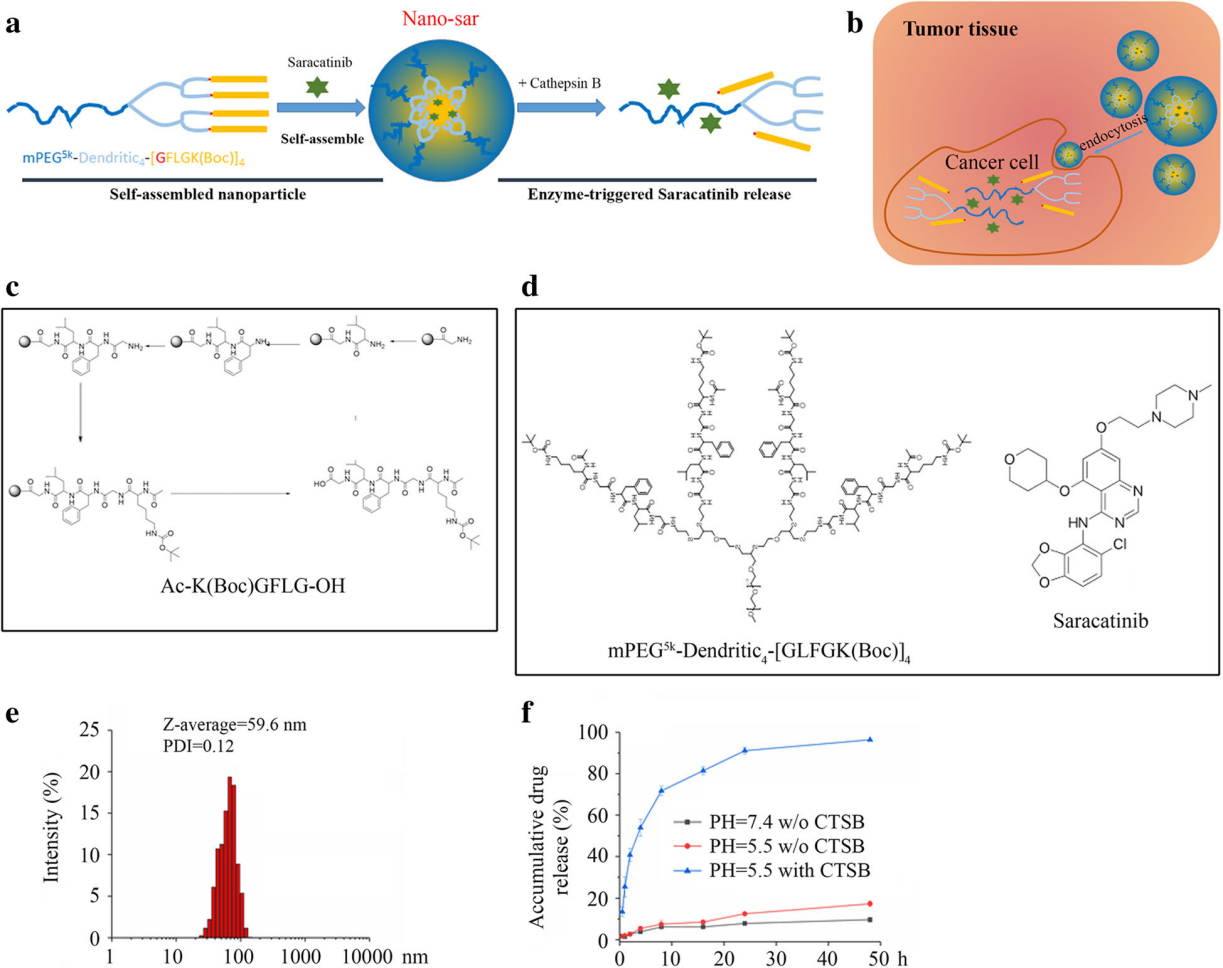
The synthesis and working principle of Nano-sar. **a** Schematic representation of the self-assemble Nano-sar and its disassembly upon CTSB digestion. **b** Schematic illustration of the working principle of Nano-sar for targeting tumor cells. **c** Solid-phase synthesis of peptide for Ac-K(Boc)GFLG-OH as a CTSB-cleavable linker. **d** Chemical structure of linear-dendritic polymeric drug carrier and saracatinib. **e** Saracatinib-loaded formulation to form nanoscale assembly as characterized by DLS. **f** The drug release profile at various conditions determined by HPLC

Saracatinib suppressed Src kinase activity more efficiently than dasatinib in HNSCC cells (Fig. 1b). Therefore, saracatinib was dissolved and encapsulated into nano-matrix with a CTSB-sensitive amphiphilic polymer. A short peptide of GFLG linker was used as hydrophobic tails, which not only facilitated to load saracatinib to obtain stable NPs but also subjected to CTSB cleavage so as to release saracatinib in tumor tissues with acidic extracellular pH feature (Fig. 3a–d). DLS study demonstrated that saracatinib was facilely encapsulated into NPs with a nanoscale size approximately 60 nm (Fig. 3e). Most importantly, Nano-sar exhibited enzyme-response drug release profile with an accelerate drug release from NPs (over 90% drug release in 48 h) in the presence of CTSB at pH 5.4, which was the active condition for CTSB (Fig. 3f). In contrast, less than 15% saracatinib was released in the absence of enzyme or at pH 7.4 (Fig. 3f). These findings suggest that Nano-sar can be stable during circulation, and saracatinib can be selectively released with the cleavage of CTSB in tumor cells.

### Nano-sar effectively inactivates Src and inhibits migration and invasion of HNSCC cells

**Fig. 4:**
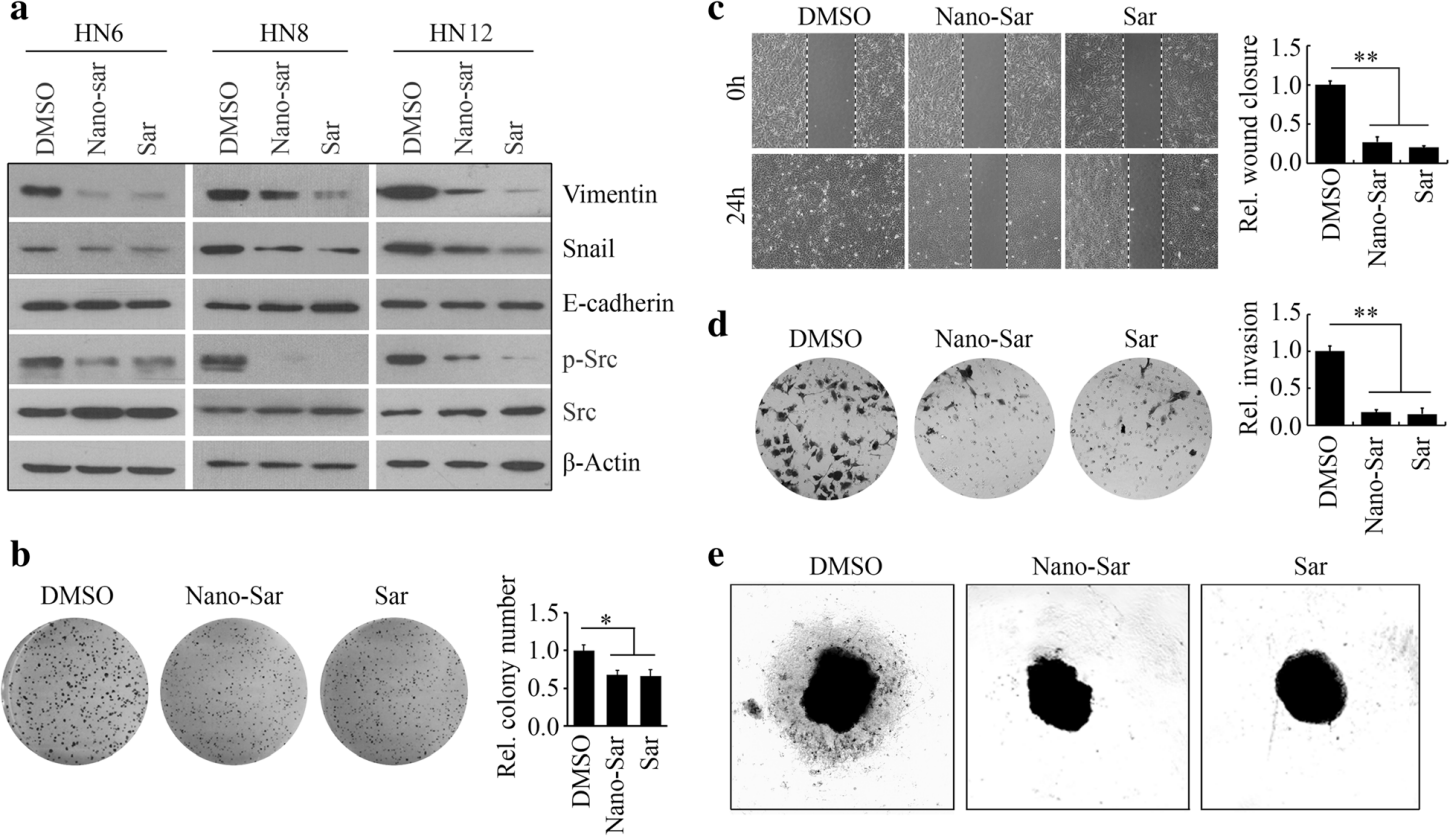
Nano-sar inhibits the Src signaling pathways, migration, and invasion in HNSCC cells. **a** The effects of saracatinib (Sar) and Nano-sar on Src phospho-activation and the downstream pathways. **b** The effects of Sar and Nano-sar on colony formation of HNSCC cells within 2 weeks. In this assay, colonies with more than 50 cells were scored and counted under the microscope. **c**, **d** The effects of Sar and Nano-sar on migration and invasion of HNSCC cells within 24 h. In **b**–**d**, the representative images and quantitative data were shown in the left and right panels, respectively. **e** The effects of Sar and Nano-sar on 3D invasion in Matrigel within 72 h. *p < 0.05; **p < 0.01

We first determined the effects of Nano-sar on Src inactivation. HN6, HN8, and HN12 cells were treated with DMSO, saracatinib, and Nano-sar, respectively. Similar to saracatinib, Nano-sar markedly inhibited the phosphorylation levels of Src, coupled with downregulation of Vimentin and Snail in HNSCC cells (Fig. 4a). Given that HN12 cells are derived from metastatic HNSCC, we further determined the anticancer effects of NP-based saracatinib in this cell line. MTS assays revealed that either the free drug or Nano-sar at the concentration of 5 μM did not exhibit the inhibitory effects on cell proliferation within 48 h after treatment, but the anti-proliferative effects of these drugs were observed from 72-h post treatment (Additional file 2: Figure S2). To confirm the long-term drug effects on cell self-renewal capability, we performed colony formation assays, which showed that the cells treated with either saracatinib or Nano-sar had lower colony forming ability than control cells (Fig. 4b). Migratory and invasive potential of HN12 cells were also significantly reduced in the presence of saracatinib, which were consistently observed in Nano-sar-treated cells (Fig. 4c, d). Next, 3D tumor spheroid invasion was assessed to confirm the results from Transwell-based assays. The process was followed over a period of 72 h as shown in Fig. 4e for HN12 spheroids, when treated with saracatinib or Nano-sar, invasion was much less pronounced compared with DMSO treatment. Nano-sar appears not to achieve obvious enhancement effects compared with the free drug in these in vitro assays, which may be resulted from quick internalization and removal of saracatinib through passive diffusion by cancer cells.

### Nano-sar exhibits superior effects on suppression of head and neck tumor metastasis than the free drug in mice

**Fig. 5:**
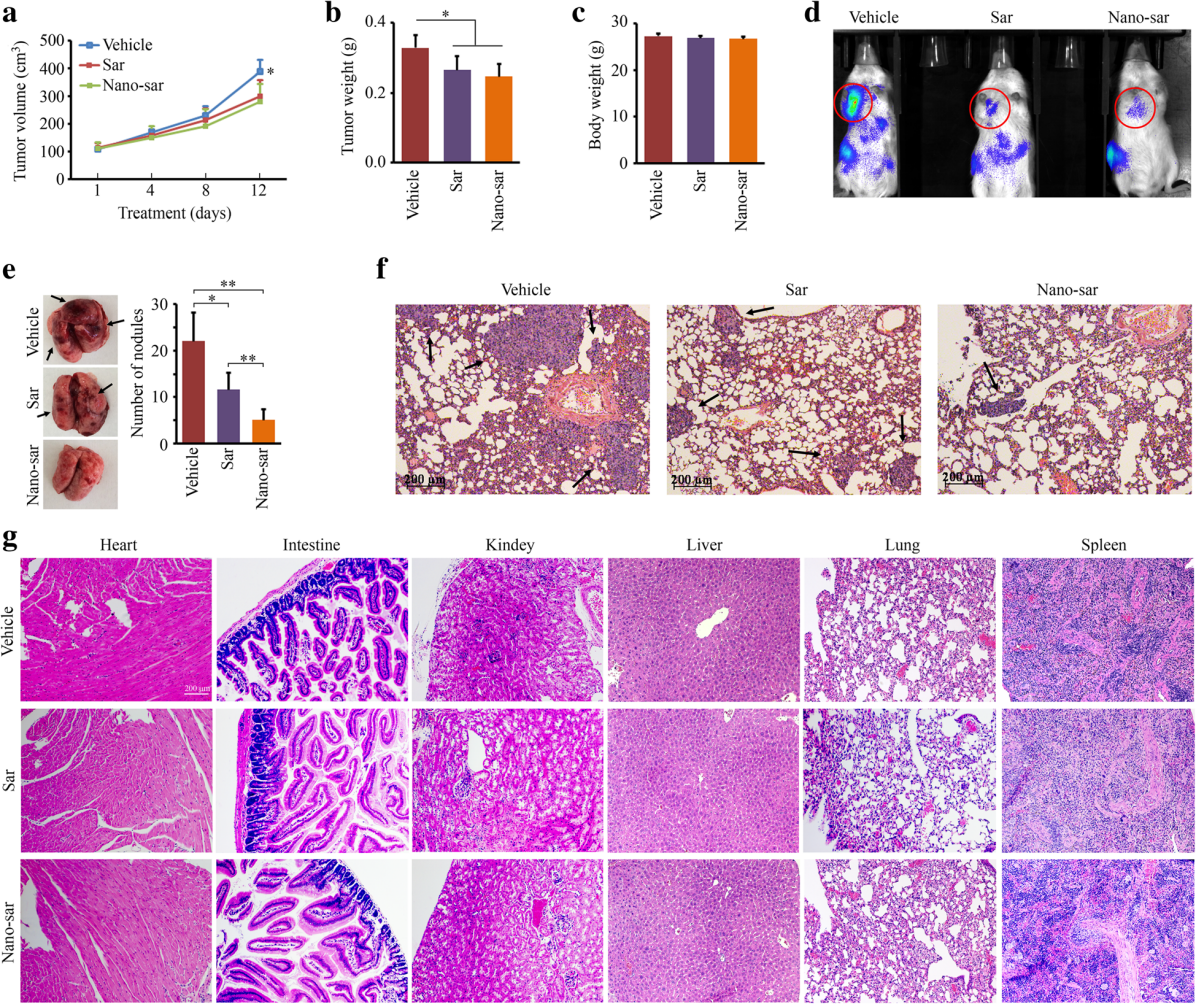
Nano-sar has superior effects on suppression of head and neck tumor metastasis than the free drug in mice. **a**–**c** Tumor growth curve, tumor weight, and body weight for mice treated with phosphate-buffered saline vehicle control, saracatinib (Sar), or Nano-sar (*n* = 5/group). **d** Tumor progression and metastasis monitored by examining bioluminescence in Xenogen IVIS-200 In Vivo imaging system. **e** The number of nodules on the lung surface. **f** Histology examination (HE staining) of the lung sections for mice treated with saline vehicle control, Sar, or Nano-sar. **g** Histology examination (HE staining) of tissues taken from major organs after therapy.*p < 0.05; **p < 0.01

The encouraging in vitro data prompted us to evaluate the efficacy of Nano-sar in mice. We have demonstrated that the subcutaneous injection of invasive cancer cells in NSG mice leads to coincident development of primary and metastatic tumors [7, 27, 38]. We then carried out the in vivo study using this cancer model. After 12 days of treatment, a reduced xenograft size and weight were observed from mice receiving saracatinib and Nano-sar, compared with those receiving vehicle (Fig. 5a, b). Saracatinib has not been shown to reduce body weight during the treatment (Fig. 5c), suggesting that this drug has no significant effects on the general well-being of the host. There was no remarkable difference in tumor growth between the mice treated with saracatinib and the mice treated with Nano-sar (Fig. 5a, b and Additional file 3: Figure S3A). However, Nano-sar suppressed tumor metastasis more efficiently compared with saracatinib as evidenced by reduced bioluminescence signal at distant sites (Fig. 5d and Additional file 3: Figure S3B) and decreased number of nodules on the mouse lung surface (Fig. 5e). Histopathological analysis further showed that treatment with Nano-sar resulted in fewer and smaller tumor foci in the lung section compared with the free drug treatment (Fig. 5f).

The promise of using Nano-sar to suppress metastasis of HNSCC encouraged us to determine the potential dose toxicity to the host. To evaluate hepatotoxicity and nephrotoxicity after drug administration, serum ALT, AST, and creatinine were measured at the endpoint of experiment. Saracatinib led to increased AST and ALT levels, but not creatinine, on mice (Additional file 4: Figure S4), which is similar to the findings from dasatinib treatment [14, 39]. Whereas, mice treated with Nano-sar did not render significant changes in all these blood biochemical indexes over a period of 12 days (Additional file 4: Figure S4), suggesting that Nano-sar at this dose does not induce notable systemic toxicity. We also collected major organs including the heart, intestine, kidney, liver, lung, and spleen from the mice receiving different treatments. Histology examinations with H&E staining on these organs did not show obviously histological difference among the groups treated with vehicle, saracatinib, or Nano-sar (Fig. 5g), indicating that saracatinib and the NP delivery system do not cause detectable systematic toxicities at pathological levels. These data suggest that Nano-sar holds potential for targeted cancer therapy in a triggered, controlled manner.

### Nano-sar suppresses metastasis through inhibiting Src-mediated EMT signaling in head and neck tumors

**Fig. 6:**
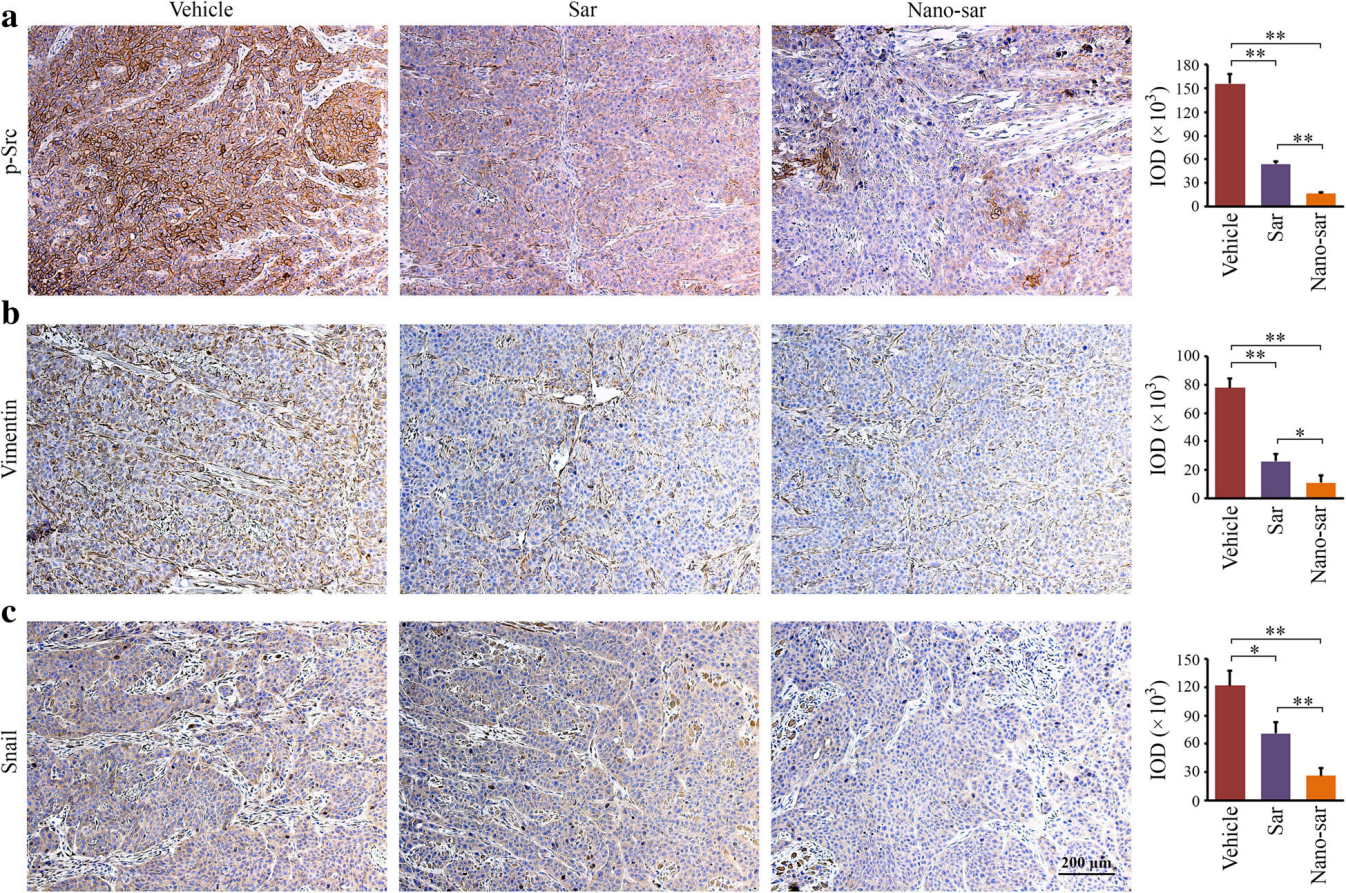
Nano-sar suppresses Src activation and the downstream pathways more efficiently in head and neck tumors than the free drug in mice. **a**–**c** The effects of saracatinib (Sar) or Nano-sar on Src signaling pathways in head and neck tumors. The representative IHC images were shown in the left panel and quantification of IHC staining with Image pro-Plus6.0 was shown in the right panel. *p < 0.05; **p < 0.01

To confirm that the effects of saracatinib on suppression of metastasis were beneficial from inactivation of Src in head and neck tumors, the xenografts from mice were immune-stained with the antibodies against p-Src, Vimentin, and Snail. Consistent with in vitro data, significantly reduced phosphorylation levels of Src were observed in tumor tissues from the mice either receiving the free drug or Nano-sar, compared with vehicle-treated mice (Fig. 6a and Additional file 5: Figure S5). Loss of protein expression of Vimentin and Snail following drug treatment was also demonstrated by IHC analysis (Fig. 6b, c). Additionally, this analysis revealed that Nano-sar suppressed Src activation and EMT-related proteins, Vimentin and Snail, more efficiently than the free drug in these head and neck tumor xenografts (Fig. 6). These observations indicate that saracatinib suppresses HNSCC metastasis, at least in part, through inhibition of Src-mediated EMT pathways.

## Discussion

Increased activity of Src is a frequent occurrence in HNSCC [10, 11]. Src acts as an integrator of divergent signal transduction pathways and promotes numerous tumor-promoting activities, including tumorigenesis, invasion, and metastasis. Therefore, inhibitors targeting Src are considered as promising drugs for cancer therapy. In this study, we demonstrate that saracatinib can effectively suppress invasion and metastasis of HNSCC, at least in part, through blocking Src-dependent Vimentin/Snail signaling. Our findings also show, for the first time, that the efficiency of tumor-responsive nano-based drug delivery system largely improves effectiveness of saracatinib in suppressing metastasis of HNSCC without systemic toxicity.

EMT is a dynamic process that endows the incipient cancer cell with invasive and metastatic properties [30]. Loss of E-cadherin-mediated cell-cell adhesion leading to detachment from neighbor epithelial cells and/or acquisition of some mesenchymal characteristics are key events of EMT [30, 40]. Src is frequently hyperactivated in cancer cells, resulting in facilitating tumor progression towards metastasis by promoting EMT [8]. For example, Src signaling has been shown to regulate E-cadherin associated EMT in pancreatic cancer cells [41]. In contrast to these studies, knockdown of Src by shRNAs or inactivation of it by small molecule inhibitors in HNSCC cells cannot affect E-cadherin levels. Instead of this, it appears that invasion repression induced by loss of Src function is resulted from downregulation of mesenchymal markers Vimentin and Snail proteins. Interestingly, all three cell lines used in this study express E-cadherin, although they show mesenchymal morphology. The possible reason is that HNSCC involves transformation of the squamous epithelial lineage, which is histologically similar to the epidermis [42]. Consistently, there were no changes in E-cadherin levels in epidermoid carcinoma A431 cells in the presence or absence of dasatinib [43]. Nevertheless, Src-mediated EMT in HNSCC cells remains to be better defined.

Surprisingly, the protein levels of total Src were increased in HNSCC cells treated with Src inhibitors, dasatinib or saracatinib, although its phosphorylation was markedly inhibited. The similar results were also observed in other studies when HNSCC cells and other types of cancer cells were treated with Src inhibitors [44–46]. Our data and previous studies suggest an unrecognized feedback mechanism for compensation of Src kinase inhibition with increased levels of Src protein expression, which maybe through downregulation of Src degradation or increase its transcription. However, the exact mechanism still needs to be deciphered.

Saracatinib, a highly selective small molecule, inhibits Src kinase activity by interfering with Src phosphorylation at tyrosine 419-human/423-mouse [47]. Preclinical studies of head and neck tumor models showed that saracatinib treatment impaired perineural invasion and cervical lymph node metastasis [48]. Here, we show a higher inhibitory rate of nano-based saracatinib in HNSCC metastasis compared with the free drug. The efficacy of Nano-sar seems to be on metastasis, which may be due to drug administration of a fixed dose within a short period of experimental time. Expansion of the time window for treatment of Nano-sar may achieve better therapeutic outcomes either on tumorigenesis or metastasis, resulting from the combined influence of Src inactivation and the tumor site-specific delivery of NPs. One of the major challenges for new therapeutics to enter the clinic remains improving their translational value to the clinical situation. We are aware that HNSCC rarely displays distant metastasis; rather, it invades and colonizes cervical lymph nodes in the clinical setting. The orthotopic mouse model of tongue tumors has been established in our group by sublingual injection of HN12 cells, but we are still facing the challenge to observe high rate of cervical metastasis in this model before tumor-bearing mice reach a moribund state. The flank model used in our study is not the best method to recapitulate HNSCC in mice; however, the analyses on it at least provide the proof of principle that the pharmacology and potency of Nano-sar is promising. Further exploration of this novel treatment in highly preclinical animal models of HNSCC is warranted.

Biocompatible and amphiphilic polymers are able to self-assemble to nanoscale formulations that possess ideal features for drug delivery, including prolonged blood circulation, high stability, and high accumulation in tumor tissues [49, 50]. As such, NPs have been explored as one of the most promising drug vehicles in the development of drug delivery system to enhance drug efficacy as well as reduce systematic toxicity. Particularly, stimuli-responsive NPs that are sensitive to biological stimuli such as pH, temperature, redox potential, and enzymes have been extensively exploited for triggered drug release. Enzymes that express at relatively low level in normal tissue but frequently overexpressed in pathological tissues appear to be an ideal stimulus. Lysosomal enzyme of CTSB, an overexpressed and secreted enzyme in tumor endothelial and epithelial cells, is one of targets that are frequently used in the development of enzyme-triggered nanomedicine. The expression of CTSB has been reported to be increased along with the cancerization in oral squamous cell carcinoma [38, 51], which is also positively associated with highly invasive and metastatic phenotypes [52]. We collected 19 primary HNSCC tissues with paired adjacent normal tissues and determined the expression levels of CTSB by real-time RT-PCR. More than tenfold higher levels of CTSB were observed in HNSCC tissues compared with paired adjacent normal tissues (data not shown), providing a strong rational basis for the design of CTSB-sensitive NP for saracatinib delivery. Given that solid tumors have an acidic extracellular environment and an altered pH gradient across their cell compartments [53, 54], the formulations of Nano-sar were designed to exploit the pH gradients that exist in tumor microenvironments. Therefore, Nano-sar can be selectively activated and release the loaded saracatinib into head and neck tumors in order to maintain effective drug levels at tumor tissues.

## Conclusion

Taking the obtained findings together, this work unveils that inactivation of Src by saracatinib can suppress invasion and metastasis of HNSCC. Several Src inhibitors have been FDA approved for the treatment of solid tumors including HNSCC. The present study provides favorable data for possible clinical application of the nano-based Src-targeting therapeutic strategy. We are convinced that with our novel drug delivery system, innovative and smart saracatinib nanomedicine can be developed for safe, efficient, and targeted cancer therapy.

## Additional files

Additional file 1: Figure S1. Either knockdown of Src by shRNA (A) or inhibition of Src phosphorylation by saracatinib or dasatinib (B) promotes reversible EMT in mesenchymal-like HNSCC cells. (DOCX 715 kb)

Additional file 2: Figure S2. MTS analysis of HN12 cell proliferation in the treatment of saracatinib and Nano-sar within 96 h. (DOCX 23 kb)

Additional file 3: Figure S3. Quantitative analysis of bioluminescence intensity from primary (A) and metastatic tumors (B). The representative bioluminescent images were illustrated in Fig. 5d. *p < 0.05; **p < 0.01. (DOCX 71 kb)

Additional file 4: Figure S4. Blood biochemical indexes of NSG mice following injection of vehicle, Sar, or Nano-sar. AST (A) and ALT (B) levels reflect hepatic functions, and creatinine (C) levels reflect nephron functions. *p < 0.05. (DOCX 33 kb)

Additional file 5: Figure S5. Mice were sacrificed on day 12 after treatment, and xenografts were dissected and removed for Western blot with the indicated antibodies. The representative image of Western blot was shown in the left panel, and quantitative data of p-Src levels were shown in the right panel (*n* = 5). 1, 2, and 3 indicate the tumor samples from three different mice. **p < 0.01. (DOCX 40 kb)

## Abbreviations

ALT: Alanine transaminase
AST: Aspartate transaminase
CML: Chronic myeloid leukemia
DMEM: Dulbecco’s Modified Eagle’s Medium
EGFR: Epidermal growth factor receptor
EMT: Epithelial-mesenchymal transition
FAK: Focal adhesion kinase
FGFR: Fibroblast growth factor receptor
GFP: Green fluorescent protein
GPCRs: G-protein-coupled receptors
HNSCC: Head and neck squamous cell carcinomas
HPLC: High-performance liquid chromatography
IGF-1R: Insulin-like growth factor 1 receptor
IHC: Immunohistochemistry
MET: Mesenchymal-epithelial transition
NP: Nanoparticle
NSG: NOD.Cg*-Prkdc*^*scid*^ *Il2rg*^*tm1Wjl*^*/SzJ*
PDGFR: Platelet-derived growth factor receptor
RTK: Receptor tyrosine kinases
SFKs: Src family of non-receptor tyrosine kinases
shRNA: Short hairpin RNA
