# Supplementary material for: Combating head and neck cancer metastases by targeting Src using multifunctional nanoparticle-based saracatinib
Source: J Hematol Oncol. 2018 Jun 20;11:85. doi: 10.1186/s13045-018-0623-3 (PMC6011403; doi:10.1186/s13045-018-0623-3)
Supplement: Supplementary file 1 — Figure S1. Either knockdown of Src by shRNA (A) or inhibition of Src phosphorylation by saracatinib or dasatinib (B) promotes reversible EMT in mesenchymal-like HNSCC cells. (DOCX 715 kb) [file 13045_2018_623_MOESM1_ESM.docx]

**
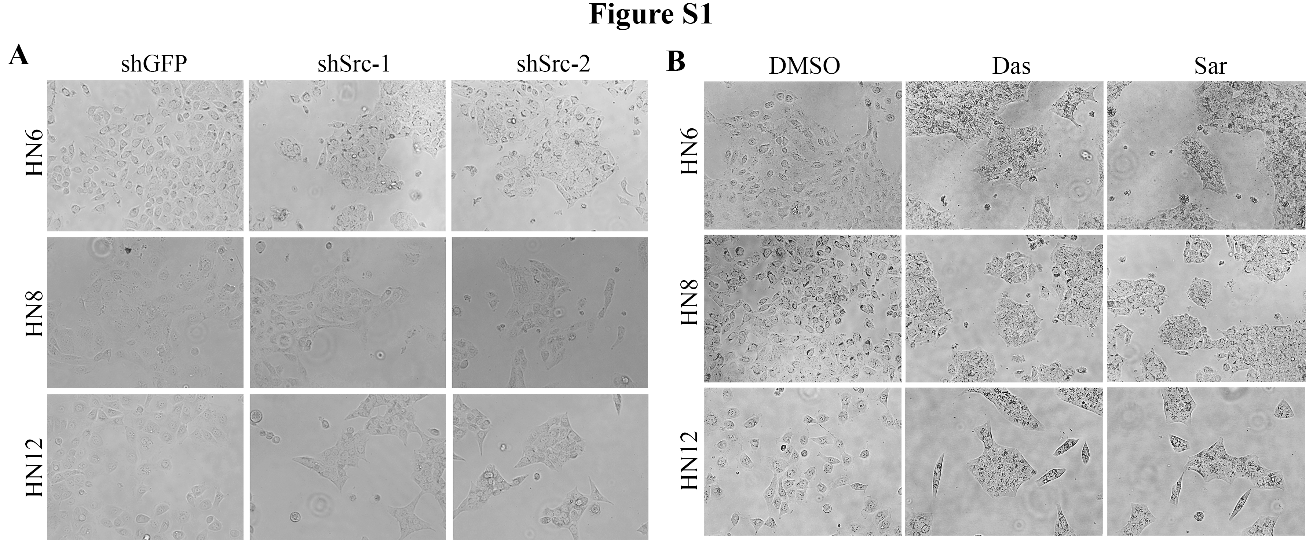
**

**Figure S1:** Either knockdown of Src by shRNA (A) or inhibition of Src phosphorylation by saracatinib or dasatinib (B) promotes reversible EMT in mesenchymal-like HNSCC cells.
