# Supplementary material for: Combating head and neck cancer metastases by targeting Src using multifunctional nanoparticle-based saracatinib
Source: J Hematol Oncol. 2018 Jun 20;11:85. doi: 10.1186/s13045-018-0623-3 (PMC6011403; doi:10.1186/s13045-018-0623-3)
Supplement: Supplementary file 2 — Figure S2. MTS analysis of HN12 cell proliferation in the treatment of saracatinib and Nano-sar within 96 h. (DOCX 23 kb) [file 13045_2018_623_MOESM2_ESM.docx]

**

**

**Figure S2:** MTS analysis of HN12 cell proliferation in the treatment of saracatinib and Nano-sar within 96 hours.
