# Supplementary material for: Combating head and neck cancer metastases by targeting Src using multifunctional nanoparticle-based saracatinib
Source: J Hematol Oncol. 2018 Jun 20;11:85. doi: 10.1186/s13045-018-0623-3 (PMC6011403; doi:10.1186/s13045-018-0623-3)
Supplement: Supplementary file 3 — Figure S3. Quantitative analysis of bioluminescence intensity from primary (A) and metastatic tumors (B). The representative bioluminescent images were illustrated in Fig. 5d. *p < 0.05; **p < 0.01. (DOCX 71 kb) [file 13045_2018_623_MOESM3_ESM.docx]

**
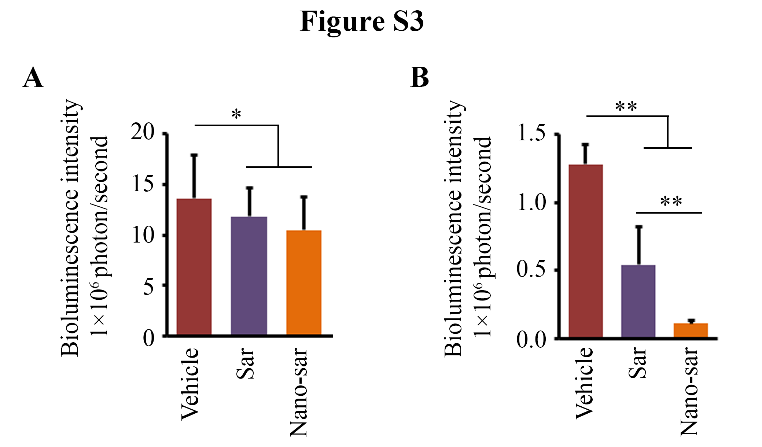
**

**Figure S3:** Quantitative analysis of bioluminescence intensity from primary (**A**) and metastatic tumors (**B**). The representative bioluminescent images were illustrated in Fig. 5D. **p* < 0.05; ***p* < 0.01.
