# Supplementary material for: Combating head and neck cancer metastases by targeting Src using multifunctional nanoparticle-based saracatinib
Source: J Hematol Oncol. 2018 Jun 20;11:85. doi: 10.1186/s13045-018-0623-3 (PMC6011403; doi:10.1186/s13045-018-0623-3)
Supplement: Supplementary file 4 — Figure S4. Blood biochemical indexes of NSG mice following injection of vehicle, Sar, or Nano-sar. AST (A) and ALT (B) levels reflect hepatic functions, and creatinine (C) levels reflect nephron functions. *p < 0.05. (DOCX 33 kb) [file 13045_2018_623_MOESM4_ESM.docx]

**

**

**Figure S4:** Blood biochemical indexes of NSG mice following injection of vehicle, Sar or Nano-sar. AST (**A**) and ALT (**B**) levels reflect hepatic functions, and creatinine (**C**) levels reflect nephron functions. **p* < 0.05.
