# Supplementary material for: Combating head and neck cancer metastases by targeting Src using multifunctional nanoparticle-based saracatinib
Source: J Hematol Oncol. 2018 Jun 20;11:85. doi: 10.1186/s13045-018-0623-3 (PMC6011403; doi:10.1186/s13045-018-0623-3)
Supplement: Supplementary file 5 — Figure S5. Mice were sacrificed on day 12 after treatment, and xenografts were dissected and removed for Western blot with the indicated antibodies. The representative image of Western blot was shown in the left panel, and quantitative data of p-Src levels were shown in the right panel (n = 5). 1, 2, and 3 indicate the tumor samples from three different mice. **p < 0.01. (DOCX 40 kb) [file 13045_2018_623_MOESM5_ESM.docx]

**

**

**Figure S5:** Mice were sacrificed on day 12 after treatment, and xenografts were dissected and removed for Western blot with the indicated antibodies. The representative image of Western blot was shown in the left panel, and quantitative data of p-Src levels were shown in the right panel (n = 5). 1, 2 and 3 indicate the tumor samples from three different mice. ***p* < 0.01.
